# An Overview: The Diversified Role of Mitochondria in Cancer Metabolism

**DOI:** 10.7150/ijbs.81609

**Published:** 2023-01-16

**Authors:** Yu'e Liu, Yihong Sun, Yadong Guo, Xiaoyun Shi, Xi Chen, Wenfeng Feng, Lei-Lei Wu, Jin Zhang, Shibo Yu, Yi Wang, Yufeng Shi

**Affiliations:** 1 Tongji University Cancer Center, Shanghai Tenth People's Hospital of Tongji University, School of Medicine, Tongji University, Shanghai 200092, China.; 2 Department of Urology, Shanghai Tenth People's Hospital, School of Medicine, Tongji University, Shanghai, China.; 3 Institute of Artificial Intelligence, Hefei Comprehensive National Science Center, Hefei, China.; 4 Xi Chen, Children's Nutrition Research Center, Department of Pediatrics, Baylor College of Medicine, Houston, TX, 77030, USA.; 5 Department of Thoracic Surgery, Shanghai Pulmonary Hospital, School of Medicine, Tongji University, 200433, Shanghai, China.; 6 Department of Pharmacology and Toxicology, University of Mississippi Medical Center, 39216, Jackson, Mississippi, USA.; 7 Department of Pathology and Medical Biology, University of Groningen, University Medical Center Groningen, Groningen, The Netherlands.; 8 Department of Critical Care Medicine, Sichuan Academy of Medical Science and Sichuan Provincial People's Hospital, University of Electronic Science and Technology of China, Chengdu, China.; 9 Clinical Center for Brain and Spinal Cord Research, Tongji University, Shanghai 200092, China.

**Keywords:** mitochondria, cancer, tumor metastasis, tumor metabolism

## Abstract

Mitochondria are intracellular organelles involved in energy production, cell metabolism and cell signaling. They are essential not only in the process of ATP synthesis, lipid metabolism and nucleic acid metabolism, but also in tumor development and metastasis. Mutations in mtDNA are commonly found in cancer cells to promote the rewiring of bioenergetics and biosynthesis, various metabolites especially oncometabolites in mitochondria regulate tumor metabolism and progression. And mutation of enzymes in the TCA cycle leads to the unusual accumulation of certain metabolites and oncometabolites. Mitochondria have been demonstrated as the target for cancer treatment. Cancer cells rely on two main energy resources: oxidative phosphorylation (OXPHOS) and glycolysis. By manipulating OXPHOS genes or adjusting the metabolites production in mitochondria, tumor growth can be restrained. For example, enhanced complex I activity increases NAD^+^/NADH to prevent metastasis and progression of cancers. In this review, we discussed mitochondrial function in cancer cell metabolism and specially explored the unique role of mitochondria in cancer stem cells and the tumor microenvironment. Targeting the OXPHOS pathway and mitochondria-related metabolism emerging as a potential therapeutic strategy for various cancers.

## Introduction

Mitochondria, known as the “powerhouse of the cell”, are one of the most crucial organelles inside the cell. They evolved from bacteria and participated in the eukaryotic cell via the process named endosymbiosis, thus improving cell energy production and generating adenosine triphosphate (ATP) via the respiratory chain. Mitochondria have their translation system and genetic material mitochondrial DNA (mtDNA) without introns which are similar to bacterial DNA. These maternally inherited mtDNAs present in multiple copies which are easily mutated due to replication errors, oxidative stress or inefficient DNA repair. Usually, cancer cells harbor somatic mutations in mtDNA and mutations of mtDNA showed advantages of tumor growth. Now the somatic mutations in mtDNA can be tracked by single-cell RNA or assay for transposase accessible chromatin (ATAC) sequencing 1.

**Figure 1:**
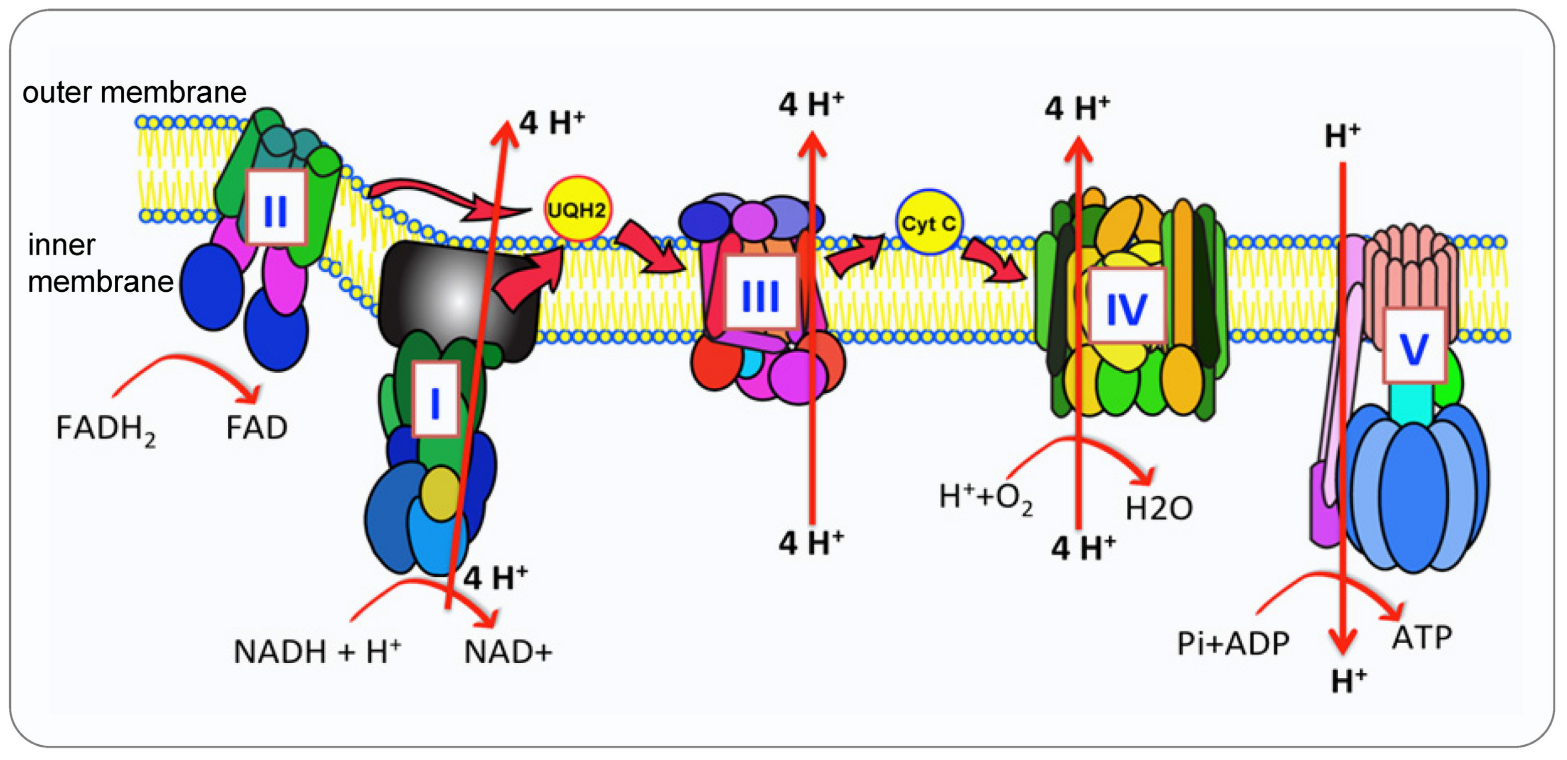
** The OXPHOS pathway.** Mitochondria produce ATP via OXPHOS carried out by ETC and synthase. In humans, the OXPHOS is comprised of five complexed embedded in the inner mitochondria membrane (IMM), the ETC (complex I-IV) are responsible for the transfer of electrons from NADH and FADH2 to oxygen which is the final electron acceptor. This electron transportation generates the electrochemical gradient across the IMM. The proton gradient drives the translocation of protons from intermembrane space back into the matrix via the ATP synthase (Complex V (CV)) that catalyzes the conversion of ADP to ATP.

Mitochondria are the metabolic hub of tumor cell proliferation, survival and metastasis due to their bioenergetic and biosynthetic function 2. The oxidative phosphorylation (OXPHOS) metabolic pathway generates ATP by transporting electrons through a series of complexes in inner membrane, known as the Electron Transport Chain (ETC) 3 (Figure 1). Proteins and complexes in the OXPHOS pathway have been identified as cancer targets, thus a myriad of inhibitors was developed in recent years 4, 5. Moreover, mitochondria are dynamic organelles, they are undergoing fusion and fission to adjust morphology to enhance cell survival. Mitochondrial trafficking is crucial during cell division, proliferation and apoptosis. To maintain the consistent dynamic condition and to keep cell homeostasis, mitochondria can execute mitophagy to degrade the damaged mitochondria 6, 7.

This review covers several fundamental functions of mitochondria, intending to introduce non-experts to understand the unique role of mitochondria and exploring the possible therapeutic target related to mitochondria.

## Mitochondrial function in cancer cell metabolism

Mitochondria are the major cellular source of NADH and house parts of the pyrimidine and lipid biosynthetic pathways, it plays various roles in cancer cell metabolism including but not limited to ATP metabolism, lipid metabolism and nucleic acid metabolism. Many different metabolites or intermediates in the tricarboxylic acid (TCA) cycle are critical in cancer cell proliferation, metastasis and apoptosis.

### Mitochondrial function in ATP metabolism

**Figure 2:**
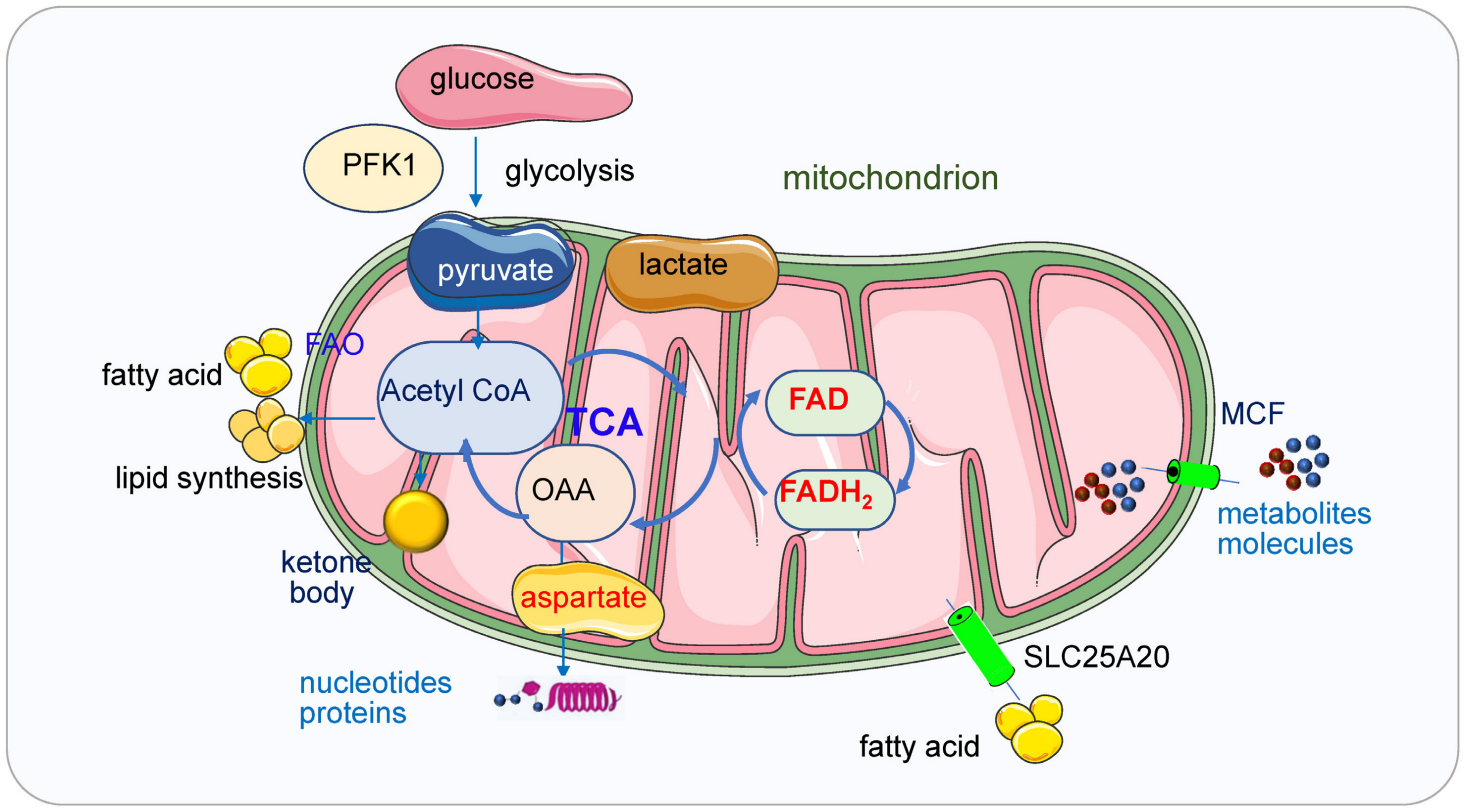
** Mitochondria in ATP, lipid, and nucleic acid metabolism.** Glycolysis regulated by PFK1 produces pyruvate and NADH from glucose and then the pyruvate enters the mitochondria under aerobic conditions and fuels the TCA cycle which produces produce metabolites. Mitochondria generate ATP through OXPHOS mainly by using pyruvate derived from glycolysis. In the absence of glucose, ATP will be produced via the degradation of fatty acids and proteins. Ketone bodies will be produced from FAO when the glucose is insufficient. Ketone bodies are generated in the mitochondrial matrix of liver cells and are subsequently exported via the blood to other organs to cover the energy demands. When the glucose transporters or ETC complexes deficiency happens, the glucose metabolism and the oxidation of pyruvate in mitochondria are bypassed, and cellular energy supply will be shifted from glucose to ketone bodies. Fatty acids are transferred from the cytosol to mitochondria by SLC25A20. In the TCA cycle, oxaloacetate generated from malic acid is transaminated to aspartate under the catalysis of transaminase and aspartate is the intermediate for synthesizing both purine and pyrimidine bases. Small metabolites or molecules are transported to the matrix of mitochondria by MCFs, which transport ADP into the mitochondrial matrix for ATP synthesis and ATP out to reach high cytosolic ATP concentrations for energy-requiring reactions.

ATP is the central energy source in the cell, it can be generated via two pathways: glycolysis in cytostome and OXPHOS in mitochondria 8. ATP production and consumption play vital roles in biosynthesis, metabolism regulation and cellular maintenance 9. Altered energy metabolism representing one of the “hallmarks of cancer” is a biochemical fingerprint of cancer cells. Glycolysis regulated by phosphorfuctokinase1 (PFK1) produces pyruvate and NADH from glucose and then the pyruvate enters the mitochondria under aerobic conditions and fuels the TCA cycle which produces produce metabolites 10 (Figure 2). These metabolites or enzymes such as isocitrate dehydrogenase (IDH), fumarate hydratase (FH), succinate dehydrogenase (SDH) and α-ketoglutarate dehydrogenase (α-KGDHC) are often mutated or deregulated in human cancers. Both glycolysis and OXPHOS pathway have been demonstrated as the targets in cancer treatment.

Due to the heterogeneity, tumors exhibit different metabolic phenotypes. In gliomas, there are both OXPHOS-dependent cells and glycolytic-dependent cells, the mechanism lies in the lactate dehydrogenase (LDH), the former expressed both LDH-A and -B isoforms whereas the latter only expressed LDH-B and LDH-B would be expected to be essential for the use of extracellular lactate to fuel cell activities 11. Even proliferating tumor cells prefer a shift towards increasing in glycolytic metabolism from OXPHOS in the presence of O_2_ (known as the Warburg effect), slow-cycling tumor cells may prefer mitochondrial respiration as a primary source of energy 12, 13. Mitochondria play essential roles in these slow-cycling tumor cells and thus targeting OXPHOS will be a promising therapy to these cancer cells.

Compared with glycolysis, OXPHOS produces ATP in a higher yield but the lower rate (30 or 32 mol ATP versus 2 mol per mole of glucose) 14. NADH and FADH2 generated from glycolysis and TCA cycle entered the matrix in mitochondria, the first four complexes embedded in the inner mitochondrial membrane transfer electrons from complex I or II to complex IV, the oxidization process of NADH and FADH coupling with the phosphorylation of ADP generate ATP, supplying energy for all the cell activities, this process is called OXPHOS 15 (Figure 1). Many diverse types of cancer cells depend on OXPHOS instead of glycolysis to obtain their ATP to promote tumorigenic potential 4, 16. Cancer cells upregulate OXPHOS and TCA cycle to obtain more ATP than their surrounding normal cells 17 and to generate resistance to chemotherapy 18. Potent OXPHOS inhibitors induce mitochondria stress from a mild state to a severe lethal energetic crisis 19.

Mitochondria generate ATP through OXPHOS mainly by using pyruvate derived from glycolysis. In the absence of glucose, ATP will be produced via the degradation of fatty acids and proteins. Ketone bodies (3-β-hydroxybutyrate, acetoacetate, acetone) will be produced from fatty acid oxidation (FAO) during fasting or prolonged exercise when the glucose is insufficient. Ketone bodies are generated in the mitochondrial matrix of liver cells and are subsequently exported via the blood to other organs to cover the energy demands 20. When the glucose transporters or ETC complexes deficiency happens, the glucose metabolism and the oxidation of pyruvate in mitochondria are bypassed, and cellular energy supply will be shifted from glucose to ketone bodies 20.

### Mitochondrial function in lipid metabolism

Mitochondria consist of around 1000 proteins and more than 400 lipids 21. Mitochondrial membrane lipids including phosphatidylethanolamine and cardiolipin are synthesized within mitochondria while other lipids are synthesized in the endoplasmic reticulum (ER) 22. Mitochondria lipid homeostasis and trafficking among different organelles inside the cell are critical to mitochondrial function 23. Mitochondria can synthesize phospholipids, phosphatidylethanolamine (PE), cardiolipin (CL) and the latter two are essential phospholipids for mitochondria respiratory functions 24, 25. Therefore, mitochondria maintain a highly active exchange of phospholipids with other cellular compartments 26.

The history of the relation between mitochondria and lipid synthesis and metabolism can be dated back to 1959 27. After lipolysis (fat mobilization), fat is decomposed into fatty acid and glycerol, then fatty acids are transferred from the cytosol to mitochondria by carnitine, which is called carnitine shuttle, fulfilled by palmitoylcarnitine transferase 1 and 2, SLC25A20 in the inner mitochondrial membrane. The β-oxidation of fatty acids takes place in mitochondria and peroxisomes 28. Free fatty acids alter respiration by providing fuel for β-oxidation, acting as weak uncouplers and activating UCP1 29.

Mitochondria associate with lipid droplets (LDs) in fat-oxidizing tissues with fatty acid storage and oxidation capacity 30, mitochondria-LD association enhances LD biogenesis and thereby protects mitochondria from lipotoxicity 31. LDs combined with mitochondria participate in metabolic regulation to mediate cellular function 32. Dgat1-dependent LD biogenesis protects mitochondrial function during starvation-induced autophagy 31, 33. mitochondria bounding with LDs are named peridroplet mitochondria (PDM) 34, it can be assessed by several quantifiable parameters such as membrane contact with an LD and adherence to an LD even after cell disruption or in restrictive purification conditions 35-37. Mitochondria in brown adipocytes continuously engage in fusion and fission activities 38. In the fission process, this PDM were segregated from cytoplasmic mitochondria and promoted triacylglyceride (TAG) synthesis 34. The segregation of PDM is achieved by keeping mitochondrial fusion proteins intact which are quite different from that in regular mitochondria whose segregation leads to disparate cristae structures, proteomes, and super-complexes. By anchoring LDs, the PDM's motility was reduced, PDM have distinct proteome and metabolic capabilities and are demonstrated as a novel node of control for lipid metabolism 39, separation of PDM from cytoplasmic mitochondria requires a modified approach. PDM can function in LDs accumulation and FAO. They are playing different roles in different physiological conditions. For example, in brown adipose tissue in mice, both the Mito-LD interaction and LD mass will be increased while FAO will be lower under the condition of thermoneutrality compared to cold exposure, thus the PDM contributes to the lipid synthesis under this condition 34, 37. In the human vastus lateralis muscle, Mito-LD interaction, LDs and FAO are higher after endurance training, thus the PDM contributes to both LDs synthesis and FAO metabolism 36. Mass spectrometry results indicate similar mitochondrial protein in CM and PDM, similar membrane potential, but the PDM have increased respiratory capacity, higher proton efflux rate and enhanced OXPHOS capacity 34. The mitochondria-lipid droplet association promotes triacyl glyceride synthesis and the peridroplet mitochondria have a unique structure, fusion dynamics, movement but reduced DPR1 recruitment and OPA1 processing. But the peridroplet mitochondria have no relation with FAO. LDs can exist in all different cell types and tissues. LDs play an important role in carcinogenesis, malignant development of cancers, and immune cells.

In addition, side products of respiratory chain such as ROS attack unsaturated lipids thus lead to lipid peroxidation (LPO), which will initiate secondary cellular responses. LPO is one of the markers for oxidative stress 40. LPO damage DNA, proteins and enzyme activity, it activates signaling pathways initiating cell death 41. Polyunsaturated fatty acids (PUFA) in the glycolipids, phospholipids and cholesterols are often the targets for PLO, among them, PUFA is one of the major targets of oxidative stress via LPO in the cellular system 42. The cardiolipin oxidation is catalyzed by cytochrome c in mitochondria, in this process, cytochrome c interacts with cardiolipin and gains peroxidase function instead of participation in electron transfer. The complex lead to cardiolipin oxidation, which is pivotal for the release of pro-apoptotic factors including cytochromes 43. Different mitochondria-targeted inhibitors of the cardiolipin oxidation pathway have been developed based on the inhibition of hydrogen peroxide or peroxidase activity of cytochrome c in this complex 44.

### Mitochondrial function in nucleic acid metabolism

Purine and pyrimidine nucleotides are required for the synthesis of RNA and DNA. The purine and pyrimidine bases are from various nonessential amino acids and methyl groups donated from the one-carbon or folate pool. DNA replication and RNA production are essential processes in cell division and proliferation, their biosynthesis is upregulated in the late G1 phase 45, 46. Nucleic acid biosynthesis is an energy-intensive process which is involved multiple metabolic pathways 47. In order to maintain sufficient energy and precursors for RNA biosynthesis, a high ATP/ADP ratio must be kept, the ATP concentration is usually stable as above 1 mM 48, 49, if the ATP level is lower than a required level, corresponding cell functions may cease or even result in necrotic cell death 50, 51. In some special stages or some high-energy requested cells such as skeletal muscle cells and cardiac myocytes, a higher ATP level (> 5mM) should be maintained to supply the materials for nucleic acid synthesis 51, 52. Cells have to adjust energy metabolism and the nucleotide biosynthetic pathways to produce enough nucleic acids for cell proliferation. Most cells synthesize nucleic acids *de novo* and mainly from glucose, glutamine and CO_2_ 53. In the TCA cycle, oxaloacetate generated from malic acid is transaminated to aspartate under the catalysis of transaminase and aspartate is the intermediate for synthesizing both purine and pyrimidine bases. Nucleotide biosynthesis can be a target for cancers too 54.

Small metabolites or molecules are transported to the matrix of mitochondria by the mitochondrial carrier family (MCF) 55. Nucleotides are one of the largest solutes to cross the inner membrane, thus the transporting process requests conformational changes of the MCF proteins 56. The ADP/ATP carrier proteins located in the inner membrane of mitochondria are the most studied MCFs 57, they transport ADP into the mitochondrial matrix for ATP synthesis and ATP out to reach high cytosolic ATP concentrations for energy-requiring reactions 56, 58. Carboxyatractyloside (CATR), a membrane-impermeable toxic inhibitor, blocks the nucleotide translocation from the inner membrane space to the matrix, the CATR and ADP binding sites from the inner membrane space partially overlap. The detailed transport mechanisms for adenine nucleotides need to be further explored 59.

### The interplay between mitochondria and glycolysis

Cancer cells exhibit heterogeneity in energy resource, some rely more on OXPHOS and some others rely on more on glycolysis. The energy resource of cancer cells may shift their reliance from one to the other. For example, loss of LUC7L2 and U1 snRNP subunits shifts the energy metabolism from glycolysis to OXPHOS 60. The recent study also demonstrated that cancer cells relying on more OPXHOS are highly expressing DNMT1 and lowly expressing NNMT, by genetically manipulating the expression of NNMT and DNMT1, the sensitivity to OXPHOS inhibitors are changed 61. Overexpression of NNMT and knockdown of DNMT1 change OXPHOS-inhibition sensitive cell line to be resistant ones, indicating their energy resource reliance shifted from OXPHOS to glycolysis 61. In the process of energy metabolic switch, the M2 isoform of pyruvate kinase (PKM2, a rate-limiting enzyme in glycolysis) interacts with the key regulator of mitochondrial fusion protein MFN2 to promote the mitochondria fusion and OXPHOS, meanwhile attenuate glycolysis 62, consistently, the activation of MFN2 interacting with PFK1 mediate PFK1 degradation and therefore suppresses glycolysis 63. In Hepatocellular carcinoma (HCC), inhibition of lysophosphatidic acid receptor 6 (LPAR6) overcomes sorafenib resistance by switching glycolysis into OXPHOS 64. Moreover, the cancer suppressor gene p53 suppresses glycolysis and promotes mitochondria oxidative phosphorylation by a series of downstream targets against the Warburg effect 65. In addition, the modification of RNA also contributes to the energy switch in cancer metastasis. In human oral cancer, the RNA modifications-5-methylcytosine (m5C)-deficient cancer cells exhibit increased glycolysis and decreased mitochondria function without affecting cell viability or primary tumor growth 66.

### Function of mitochondrial metabolism in cancer

**Figure 3:**
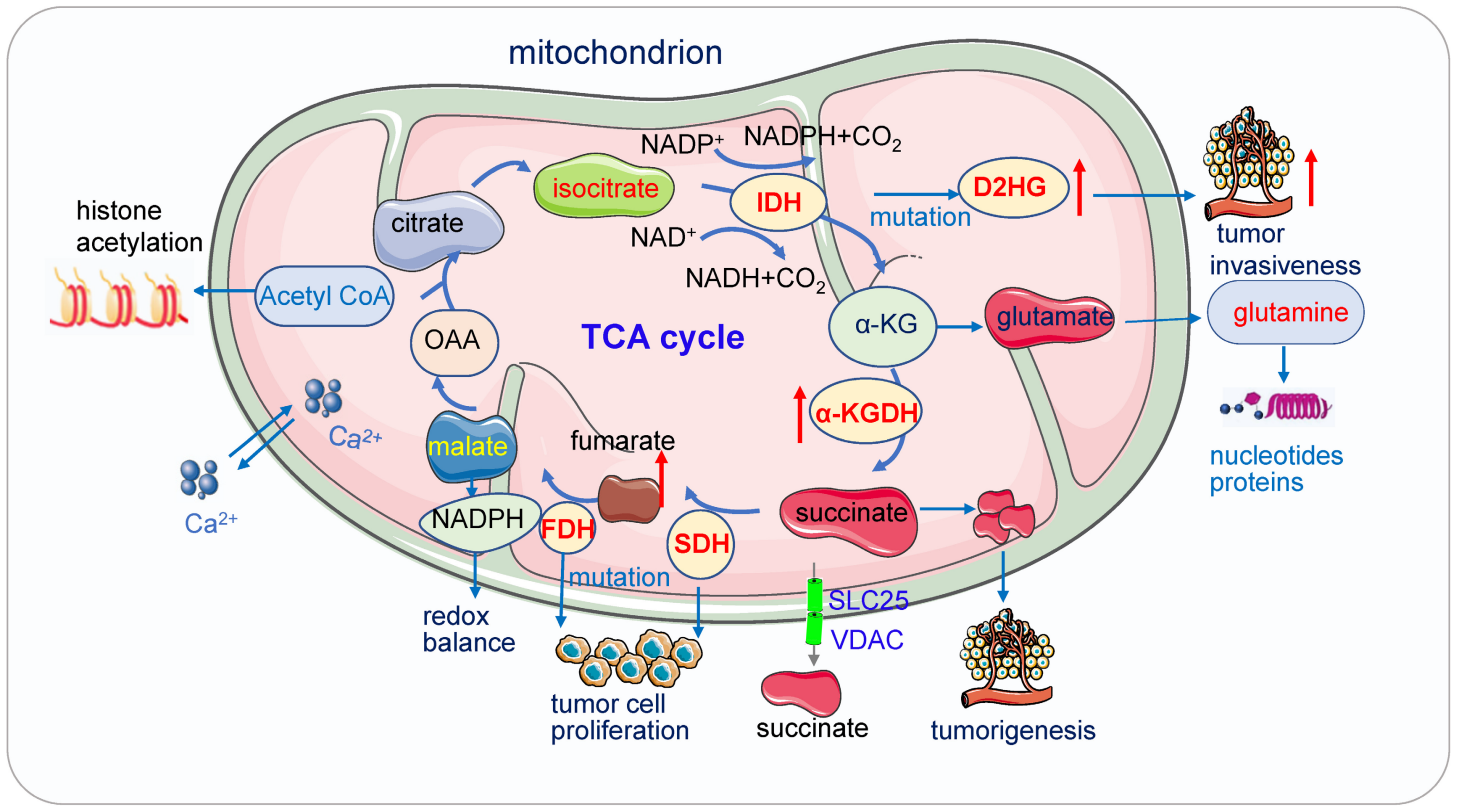
** Mitochondria function in cancer metabolism.** 1) Mutation of enzymes in TCA cycles such as mutation of IDH, SDH leads to high concentrations of D2HG, fumarate and succinate accumulation promoting tumor cell proliferation and progression. These enzymes also serve as the driver of human cancer. The metabolic dysregulation is not only a consequence of oncogenic transformation but also that it drives cancer. 2) Acetyl-CoA which is the most important substrates for acetylation modification, can be derived from glucose (pyruvate oxidation), fatty acid, and amino acid catabolism, it is the key indicator of cell metabolism and links metabolism, signaling, and epigenetics. 3) Succinate exists intracellular (cytosol and mitochondria) and extracellular, its efflux from mitochondria to the cytosol relies on SLC25 and VDAC.

Mitochondrial metabolism plays an essential role in a variety of physiological and pathological processes (Figure 3). It is crucial for tumor proliferation, survival, metastasis and drug resistance 67, 68.

#### Acetyl-coenzyme A

Acetyl-coenzyme A (Acetyl-CoA), the most important substrates for acetylation modification, can be derived from glucose (pyruvate oxidation), fatty acid, and amino acid catabolism, it is the key indicator of cell metabolism and links metabolism, signaling, and epigenetics 69-71. Fluctuations of Acetyl-CoA have been demonstrated to be closely associated with changes in overall histone acetylation and gene expression 70, 72. Under the physiological stage, dynamic histone modifications have important roles in Maternal-to-zygotic transition (MZT). In mouse embryos, H3K4me3 and H3K27ac module MZT process 73. In pigs, Acetyl-CoA synthases are essential for maintaining ATP and histone acetylation in porcine early embryos under metabolic stress during Zygotic genome activation (ZGA) 74. During early embryogenesis, H4K16ac is maintained from oocytes to fertilized embryos in Drosophila and mammals, providing an instructive function to the offspring by priming gene activation 75. The level of Acetyl-CoA and histone acetylation also controls the early differentiation of embryonic stem cells, inhibition of acetyl-CoA or its upstream causes differentiation of pluripotent cells, while inhibition of its downstream delays differentiation 76, 77. Cooperation between lncRNA DIGIT and BRD3 at the regions of H3K18ac regulates the transcription factors which drive endoderm differentiation 78. In embryonic neurol stem cell (NSC), histone acetylation plays important roles in NSC differentiation, TP53 inducible glycolysis and apoptosis regulator (TIGAR) promotes NSC differentiation through upregulating Acetyl-CoA 79. Under the pathological state, acetyl-CoA play important roles in tumor migration. For example, in glioblastoma, it promotes the expression of cell migration and adhesion-related genes by controlling Ca^2+^ and nuclear factor of activated T called signaling 72, 80. In pancreatic carcinogenesis, histone acetylation facilitates cell plasticity and proliferation 81. And in the non-small-cell lung cancer (NSCLC), acetyl-CoA carboxylase (ACCS) catalyzes the ATP-dependent carboxylation of acetyl-CoA to form malonyl-CoA, it is required for de novo fatty acid synthesis needed for tumor growth and viability. Inhibition of ACCS suppresses fatty acid synthesis and thus suppress tumor growth 82, ACCS is a potential cancer target for cancer therapy. Acetyl-CoA synthetase 2 (ACSS2) contributes to cancer cell growth under low-oxygen and lipid-depleted conditions by promoting acetate utilization 83. In hepatocellular carcinoma (HCC) metastasis, acyl-CoA thioesterase 12 (ACOT12) links the alteration of acetyl-CoA with HCC metastasis by suppressing HCC metastasis both *in vitro* and *in vivo* 84.

#### Citrate

Citrate in mitochondria is oxidized in the TCA cycle to produce ATP, it is important for lipid synthesis and epigenetic regulation; cancer cells import extracellular citrate to support their growth 85. Citrate can also be transported to the cytosol and cleaved by citrate lyase to regenerate acetyl-CoA and oxaloacetate 86, 87. Manipulating citrate level affects both cancer and immune cells thus modulating cancer cell apoptosis and immune responses 85. Citrate level keeps low in highly proliferative cancer cells, it is an activator of gluconeogenesis and an inhibitor of glycolysis 88, 89. For cancer cells relying more on glycolysis, this low level of citrate help to sustain the Warburg effect, but for others relying more on OXPHOS, more citrate is produced from FAO but it is converted to acetyl-CoA and oxaloacetate quickly 87. Elevation of intracellular citrate level arrests glycolysis, proliferation, dedifferentiation, and aggressiveness of cancer cells 90. In prostate cancer, citrate activates the autophagic death of cancer cells by downregulating CaMKII/AKT/mTOR pathway 91. In addition, citrate inhibits the proliferation of multiple cultured cancer cells such as ovarian, mesothelioma, pancreas, lung, stomach, and melanoma, indicating that citrate might be a promising anti-tumor target.

#### Isocitrate

Isocitrate is the product of aconitase and the substrate of isocitrate dehydrogenase, it is an intermediate metabolite in the citric acid cycle found both inside the mitochondria and outside in the cytosolic shunt. Treatment of isocitrate may enhance breathing and render it more resistant to hypoxic insult and increase the amplitude of ventilation *in vivo* in normoxia, indicating isocitrate is helpful in avoiding the failure of gasping generation and autoresuscitation in pathological conditions. The application of isocitrate showed a significant and persistent attenuation for anemia of inflammation in a mouse acute and severe anemia of inflammation (AI) and in a rat arthritis model of moderate chronic AI 92, 93.

Isocitrate is oxidized by the enzyme IDH, where carbon dioxide is liberated and 2-oxoglutarate (2OG, also known as α-ketoglutarate) is the oxidized product, it converts isocitrate to α-KG. IDH can be inside mitochondria in a nicotinamide adenine dinucleotide (NAD)^+^-dependent manner and nicotinamide adenine dinucleotide phosphate (NADP)^+^-dependent manner, it can also be formed in the cytosol in an NADP^+^-dependent manner 94. IDH1 and IDH2 impair the production of 2OG and reduce NADPH from isocitrate, meanwhile they oxidize NADP^+^ and promote 2OG to D-2-hydroxyglutarate (D-2HG) 95. The elevated d-2HG is a biomarker for many cancers 96. *IDH1* is the most frequently mutated gene in cancer. In low-grade glioblastoma (LGG), more than 80% of *IDH* mutations occur in the* IDH1* gene, being dominated by R132H *IDH1* 97.

Moreover, IDH mutations are associated with the altered IL-1β responses in acute myeloid leukemia (AML) 98. Mutations in *IDH1* and *IDH2* induce epigenetic and transcriptional reprogramming, and differentiation bias and promote the development of a number of malignancies 99, 100 including but not limited to chondrosarcoma, AML 101, 102, cholangiocarcinoma 103, prostate cancers 104, angioimmunoblastic T-cell lymphomas 105, myeloid neoplasia 106. Thus, inhibition of IDH1 and IDH2 variants is being pursued as a medicinal chemistry target 107.

#### α-ketoglutarate

α-KG is a membrane-impermeable and key endogenous metabolite in the TCA cycle, its level changes upon fasting, exercise and aging 108. Level of α-KG affects cell metabolism, collagen synthesis, epigenetic regulation, stem cell proliferation and immune response 109. Intracellular α-KG inhibits starvation-induced autophagy and it has no direct respiration-inhibitory effect 110. α-ketoglutarate can extend lifespan in mice by suppressing chronic inflammation via the induction of IL-10 by dietary 108.

α-KG is an important cofactor for histone demethylase including Jumonji C (JmjC) and TET, it plays important roles in methylation modification 111, 112. In age-related osteoporosis, α-KG decreases H3K9me3 and H3K27me3 and upregulates BMP signaling and Nanog expression, resulting in rejuvenating mesenchymal stromal/stem cells (MSCs) and ameliorating age-related osteoporosis 113. α-KG directly regulates signal pathway, in colorectal cancer (CRC), α-KG attenuates the Wnt signaling pathway by promoting hypomethylation of DNA and histone H3K4me3 and drives differentiation of CRC cells, identifying α-KG as a potent antineoplastic metabolite for potential differentiation therapy for CRC patients 114. In p53-deficient pancreatic ductal adenocarcinoma (PDAC), the addition of cell-permeable α-KG increased cell differentiation and decreased tumor-cell fitness, indicating the role of α-KG in linking p53 to cell fate during tumor suppression 115. α-KG also plays important roles in the immune system, α-KG suppresses M1 macrophage activation but promotes M2 macrophage activation to exhibit anti-inflammatory effects by mediating metabolic and epigenetic reprogramming 116.

α-KG is catalyzed by α-ketoglutarate dehydrogenase (α-KGDH) and metabolized into succinyl-CoA, this GDH-mediated α-KG can inhibit IKKβ activation and block nuclear factor κB (NF-κB) activation, promote glucose update and cell survival by upregulating GLUT1, thereby accelerating gliomagenesis 117. α-KGDH inhibition increases α-KG levels and leads to DNA demethylation and finally impairs cell migration in breast cancer-associated lung metastasis 118.

#### Succinate

Succinate is a key modulator of cell metabolism in hypoxic response, an important player in tumorigenesis, protein succinylation and inflammatory signal 119. Succinate exists intracellular (cytosol and mitochondria) and extracellular, its efflux from mitochondria to the cytosol relies on solute carrier family 25 (SLC25) and the voltage-dependent anion channel (VDAC) 120. Succinate can be generated via three pathways: the reductive branch of the TCA cycle, the glyoxylate pathway, and the oxidative TCA cycle.

Succinate also acts as an inflammation signal 119, 121, a key player in macrophage activation 122, 123. It inhibits transcription factor hypoxia-inducible factor-1α (HIF-1α) prolyl hydroxylases in the cytosol, leading to stabilization and activation of HIF-1α via succinate receptor in specific tumors and in activated macrophages and stimulates dendritic cells 121, 124. Succinate is an extracellular metabolic stress signal sensed by the mainly Gi-coupled succinate receptor SUCNR1, regulating the transcription of human M2 macrophages 125. Tumor cells dictate anti-tumor immune responses by altering pyruvate utilization and succinate signaling in CD8^+^ T cells 126.

Succinate and other metabolites with similar structures, such as D-2HG and fumarate, are considered oncometabolites 127. Accumulation of succinate promotes immune function and tumorigenesis 128, circulating succinate has emerged as a promising biomarker in chronic metabolic diseases. Elevated succinate levels within the gut lumen association with microbiome disturbances (dysbiosis) and in patients with inflammatory bowel disease (IBD) and animal models of intestinal inflammation 120. Physiologically, succinate activates the receptor GPR91 identified in the bladder and it is essential to bladder structure and contraction 129.

SDH, also known as mitochondrial Complex II, is a mitochondrial enzyme participating in both the citric acid cycle and the electron transport chain. It oxidizes succinate to fumarate in the TCA cycle and oxidizes ubiquinone to ubiquinol in the mitochondrial ETC 130. Localized in the inner membrane of mitochondria, Complex II holoenzyme consists of four subunits, SDHA, SDHB, SDHC, and SDHD, and two assembly factors, SDHF1 and SDHF2 131.

SDH has now been defined as a tumor suppressor and succinate an oncometabolite 132. SDH deficiency leads to the accumulation of succinate 133, 134, which inhibits α-KG dependent dioxygenase family enzymes. These enzymes include around 60 members and regulate key aspects of tumorigenesis such as DNA and histone demethylation, hypoxia responses, and m6A mRNA modification 127. SDH deficiency changes cellular metabolism, such as the demand for extracellular pyruvate and the biosynthesis of aspartate 135, 136. SDH-deficient cells also increase activities in glycolysis, lactate production, and pentose phosphate pathways 137. Dysfunction of the SDH impairs mitochondrial activity, ATP generation and energy hemostasis. Increased mitochondrial oxidation of succinate by SDH and an elevation of mitochondrial membrane potential (MMP) induces mitochondrial ROS production 122. Functional SDH deficiency is a common adverse feature of clear cell renal cell carcinoma (ccRCC) 138. SDH gene germline mutations lead to SDH-deficient renal cell carcinoma 139. Moreover, SDH acts as a key regulator in neurodegenerative disorders 140, neuroendocrine tumors 141, Chronic obstructive pulmonary disease 142. In addition, SDH is critical for metabolic and epigenetic regulation of T cell proliferation and inflammation, SDH deficiency induced a proinflammatory gene signature in T cells and promoted T helper 1 and T helper 17 lineage differentiation 143.

#### Fumarate

Fumarate is generated in the TCA cycle by the SDH-catalyzed dehydrogenation of succinate, acting as a bona fide oncogenic molecule and a key activator of a variety of oncogenic cascades 144. Fumarate will be accumulated when FH is deficient, which leads to high concentration fumarate in multiple subcellular compartments and the extracellular microenvironment, affecting the balance of multiple enzymatic reactions and induction of tumorigenesis. The loss of FH and the accumulation of fumarate elicit the pro-oncogenic signals which contribute to the transformation of normal cells into tumor cells.

FH is distributed in both cytosol and mitochondria; the mitochondrial FH is part of the TCA cycle and it catalyzes the reversible hydration of fumarate to malate. The mutation of FH has been identified in a variety of cancers. FH-deficient cells respond to mitochondrial impairment by a series of compensatory metabolic changes: they increase their glycolytic pathway with the increase of glycolytic-related gene transcription and the inhibition of pyruvate dehydrogenase (PDH); the glutamine is converted to α-KG and eventually to citrate via reductive carboxylation. FH-deficient cells harbor lower fumarate and preserve Aconitase 2 (ACO2) function to promote tumor progression.

The FH deficiency also affects epigenetics. The loss of FH causes hypermethylation in the promoter of the tumor suppressor cyclin-dependent kinase inhibitor 2A (CDKN2A), the hypermethylation of CDKN2A is a predictive factor for unfavorable prognosis of various cancers 145. It also causes the hypermethylation and suppression of MIR200 and leads to an epithelial-to-mesenchymal transition (EMT), which promotes tumor metastasis 146. The accumulation of fumarate inhibits JmjC-KDMs and increases the methylation of H3K4, H3K27 and H3K79, which promote gene transcription 147.

#### Malate

Malate is synthesized from fumarate by fumarase and further oxidized to oxaloacetate by malate dehydrogenase (MDH) with the accompanying reduction of NAD+. The metabolic imbalance results in the impairment of either mitochondrial and/or cytoplasmic metabolism. The oxidation of cytosolic NADH by the mitochondria requires malate-aspartate shuttle 148, by which cells maintain a balance of metabolic intermediates between the cytosol and the mitochondria to enable lactate metabolism to continue. This shuttle controls NAD^+^/NADH homeostasis to maintain the activity of mitochondrial MDH and promote L-lactate oxidation in mitochondria 149. Inhibition of the malate-aspartate shuttle leads to the decrease of ATP production in glioma cells 150. The major function of the shuttle in cancer cells is to maintain glycolysis, instead of increasing mitochondrial energy metabolism.

MDH reversibly converts malate to oxaloacetate using NADH and serves as important oxidoreductase in several metabolic pathways. MDH has two isoenzymes, MDH1 in cytosol and MDH2 in mitochondria. Both are closely associated with cancers. The mutation of MDH2 destroys the TCA cycle and affects energy metabolism, leading to mitochondria dysfunctional diseases such as epilepsy, hypotension etc. The upregulation of MDH2 increase energy metabolism and the deficiency of MDH2 reduces the production of ATP and the augment of ROS. MDH2 is highly expressed in many types of cancers, MDH2 concentration was higher in early-stage NSCLC patients compared with that in controls, therefore, MDH2 can be a potential biomarker for early detection of NSCLC 151.

#### Oxaloacetate

Oxaloacetate can be generated from malate, pyruvate, phosphoenolpyruvate and aspartate. It plays important roles in cancers and other diseases by modulating cell metabolism. OAA is a competitive inhibitor of LDHA, high pyruvate kinase M2 (PKM2) activity reduces LDHA activity via upregulating cytosolic OAA in cancer cells, the elevated PKM2 increase the *de novo* synthesis of OA and inhibit LDHA in cancer cells 152. In KRAS-mutated cancer cells, inhibition of glutamate oxaloacetate transaminase 1 (GOT1) increases the sensitivity to glucose deprivation, GOT1 is critical to provide oxaloacetate at low glucose to maintain the redox homeostasis 153. Furthermore, treatment of oxaloacetate in human hepatocellular liver carcinoma cell inhibit the glycolysis and enhance the OXPHOS, leads to cancer cell apoptosis 154. In addition, oxaloacetate has been demonstrated playing a neuroprotective role for acute ischemic stroke since it reduces the brain and blood glutamate levels when the blood-resident enzyme glutamate-oxaloacetate transaminase is activated 155. It also protects liver from warm ischemia injury by improving cellular energy metabolism 156.

## Mitochondrial function in cancer stem cells

Cancer stem cells (CSC) or tumor-initiating cells, characterized by the ability to self-renew, are a small subset of cancer cells responsible for tumor initiation, maintenance, growth, metastasis, recurrence and drug resistance. Mitochondria play crucial roles in cancer stem cells in different aspects such as cell metabolism, drug resistance, apoptosis etc. Metabolic reprogramming through the regulation of mitochondrial activities is a key feature of CSCs 157. Inhibition of mitochondrial translation and mitochondrial fission, targeting the OXPHOS pathway or the related key genes are potent therapeutic strategies in cancer stem cells 158. Therefore, mitochondria have been seen as a new therapeutic target for eradicating cancer stem cells 159.

### CSCs rely on more on OXPHOS

Increasing evidence had demonstrated that a myriad of CSCs relies highly on OXPHOS for obtaining energy 160. They are less glycolytic, consuming less glucose and producing less lactate while maintaining higher ATP levels than their differentiated version and surrounding bulk tumor cells 161. CSCs possess high mitochondrial membrane potential and elevated mitochondrial gene expression, over-expressed PGC-1α 162. In pancreatic ductal adenocarcinoma (PDAC) cancer stem cells, the utilization of OXPHOS increased tumorigenic potential and pluripotency gene expression, enhanced invasiveness and upregulated immune evasion properties 163.

Inhibition of OXPHOS in CSCs induces cell death, in chronic myeloid leukemia (CML), the combination treatment with imatinib (a tyrosine kinase inhibitor) and tigecycline (an antibiotic) that inhibits mitochondrial protein translation, selectively eradicates CSCs both *in vitro* and in a xenotransplantation model of human CML 164. In AML, it also promotes cell differentiation 164-166. Various OXPHOS inhibitors have been demonstrated as cancer stem cell killers. For instance, Targeting Co-enzyme Q10 of mitochondrial complex III by atovaquone 167, targeting complex I by Rotenone 159, and targeting complex V by Gboxin 13 eradicate CSC effectively.

### CSCs possess higher PGC-1α

PGC1-α, the transcription co-activator peroxisome proliferator-activated receptor gamma co-activator 1α, acts as a master regulator of mitochondria *de novo* synthesis, and regulates different energy-producing metabolic processes including OXPHOS. It has been demonstrated to be overexpressed in circulating tumor cells. High expression of PGC-1α enhances mitochondrial biogenesis, strong antioxidant activity and fewer ROS, increase OXPHOS and promote cancer cells' invasion and metastasis 168, 169. Suppression of MYC and subsequent increase of PGC-1α were identified as key determinants for the OXPHOS dependency of CSCs, which was abolished in resistant CSC clones 170.

### CSCs exhibit lower ROS

ROS as the side product of the respiratory chain has been applied in radiation therapy, which causes DNA damage directly by ionization or indirectly through the generation of ROS to destroy cancer cells 171. CSCs possess a more powerful antioxidant defense system which can counteract and scavenge ROS to keep their low ROS level and maintain their stemness and tumorigenesis 172. In glioblastoma stem cells, the generation of ROS in mitochondria can be suppressed by the combination of Prohibitin and PRDX3, which lead to the degradation by the ubiquitin-proteinase system 173.

### Mitochondria fission and fusion affect CSCs' cell fate

Mitochondria are dynamic organelles frequently undergoing fission and fusion to maintain their morphology and regulate their function. Mitochondrial morphology and function contribute to a stem-like phenotype of cancer cells 174, implying that mitochondrial fission and fusion are critical players in CSC behavior. Mitochondrial fission may play a role in the removal of damaged mitochondria. High levels of mitochondrial fission activity are associated with high proliferation and invasiveness in some cancer cells and with self-renewal and resistance to differentiation in some stem cells 175. Mechanistically, the FOXO and Notch signaling converge on the regulation of mitochondrial fission and in turn, the mitochondrial fission provokes the CSC differentiation into the bulk cells, thus the mitochondria morphology affecting cell fate of CSCs 176.

The exogenous mitochondrial transfer and endogenous mitochondrial fission facilitate the AML resistance to OXPHOS inhibition 177. The processes controlling mitochondria dynamics are mediated by specific proteins such as mitochondrial out membrane fusion protein MitoPLD, inner membrane fusion protein OPA1, mitochondrial fission protein Drp1 and its receptors FIS1, MID49, MID51, etc. 6, 178. The mitochondria fission factor FIS1 augments mitophagy in lung CSCs to maintain their stemness 179. Mitochondria fission has been treated as a driver of stemness in tumor cells. Drp1, the essential mediator of mitochondrial fission, has been found to be hyperactivated in brain tumor-initiating cells and its phosphorylation regulates mitochondrial morphology and stem cell marker expression 180. mDIVI1, an inhibitor of the mitochondrial fission protein DRP1, inhibits 3D tumor sphere-forming capacity, cell migration and stemness-related signaling in breast cancer cells via inducing the loss of oxidative phosphorylation 181.

By asymmetric division, stem cells generate two daughter cells in different traits. Daughter cells that received fewer old mitochondria maintain stem cell traits. The failure to asymmetrically apportion old mitochondria in a single division can cause a persistent loss of stemness in stem cells and cancer stem cells 182.

### Mitophagy in CSCs

Mitophagy is an evolutionarily conserved cellular process, it is critical in maintaining mitochondrial integrity and cell homeostasis by removing damaged or surplus mitochondrial fragments to the lysosome for degradation 183. Mitophagy is essential in the differentiation and in the acquisition of “stemness” in cancer stem cells 184, 185. It is balanced with mitochondrial biogenesis to maintain mitochondrial homeostasis in CSCs and promote CSCs plasticity for better adaption in tumor microenvironment (TME) 186, 187. Mitophagy also contributes the energetic and metabolic shift from glycolytic phenotype into an OXPHOS state of CSCs via their dynamic adjustment 183, 184. PINK1-dependent mitophagy driving mitochondrial rejuvenation affects pluripotency and differentiation state in stem cells 188. In Pancreatic cancer stem cells (PaCSC), ISG15 and ISGylation are required for mitophagy and metabolic plasticity, loss of ISG15 lead to increased accumulation of dysfunctional mitochondria, reduced OXPHOS and impaired mitophagy and finally negatively impacts PaCSC stemness 189. In HCC, mitophagy induction increases the hepatic CSC population while its inhibition decreases it 190, mitophagy promotes transcriptional activation of NANOG to maintain the self-renewal and cell stemness 190, 191. Mitophagy is also required for self-renewal of human acute myeloid leukemia stem cells 192. In glioblastoma CSCs, the platelet-derived growth factor (PDGF) signaling inhibits the activation of mitophagy and promotes cancer stem cell maintenance by inducing N^6^-methyladenosine (m^6^A) accumulation 193. Furthermore, CSCs may also reply on mitophagy to keep low ROS levels and prevent the activation of programmed cell death 194. However, an excessive rate of mitophagy flux can also confer chemoresistance 195. Therefore, the role of mitophagy in CSCs may be more important than previously recognized.

## Mitochondria in the tumor microenvironment (TME)

TME comprises various cell types including tumor cells, immune cells and the surrounding environment of tumors. TME plays pivotal roles in tumor imitation, progression and metastasis 196. The heterogeneous TME requests different oxygen supply which leads to the heterogeneity of mitochondrial distribution 68. T cell infiltrating tumors exhibit decreases in mitochondrial function and mass, this TME represses T cell mitochondrial biogenesis and T cell oxidative metabolism 197. The hypoxia microenvironment of tumors requests mitochondria to adjust ETC activity, ROS production and reduce the TCA cycle to survive and proliferate in oxygen insufficiency.

### ROS in cancer cell metabolism

Mitochondria generate ROS from complex I and complex III at the inner mitochondrial membrane during oxidative phosphorylation, this can be identified by inhibitors of the mitochondrial respiratory chain such as Rotenone and Antimycin A as well as the substrates for various complexes such as pyruvate or succinate 198. Excessive ROS quantity induces mitochondria dysfunction by inducing a significant decrease in mtDNA-encoded gene transcripts for respiratory complexes I, III and IV. mtDNA is sensitive to oxidative damage due to its low repair capacity and close relation with the electron transfer chain, it has been one of the main targets of ROS 199. mitoTEMPO, a mitochondria-targeted superoxide dismutase mimetic, decreases mitochondrial ROS production and reduces hypertension 200. Mitoquinone, another mitochondrial-targeted antioxidant, can improve mitochondrial function and attenuate redox-related cardiomyopathies, but in cancer cells, it could also lead to ROS production, membrane depolarization, and apoptosis 201.

Mitochondrial ROS also oxidizes proteins in the ETC and leads to a decrease in mitochondrial respiration and finally causes related signaling pathway activation 202, 203. ROS concentration inside the cell is modulated and tightly controlled to remain in a concentration range compatible with cell proliferation and to avoid ROS overproduction, provoking damage to mitochondria and extra-mitochondrial macromolecules and eliciting cell death 10, 204. Cancer cells are able to reduce ROS quantity to survive and invade surroundings. Antioxidant proteins such as peroxiredoxins were specifically upregulated in lung metastatic breast cancer cells 205, PRDX2 had been demonstrated to protect metastatic cancer cells from oxidative stress 206.

The interaction of ROS and autophagy play important roles in both cell damage and cell survival. ROS may regulate autophagy at transcriptional and post-transcriptional levels via various signal pathways such as OS-FOXO3-LC3/BNIP3-autophagy, ROS-NRF2-P62-autophagy, ROS-HIF1-BNIP3/NIX-autophagy and ROS-TIGAR-autophagy 207. By contrast, in tumor cells, autophagy can reduce the excessive production of mitochondrial or cytosolic ROS 208. Oxidative damage leads to the production of ROS, but meanwhile also initiates autophagy to clear the increased ROS.

ROS can modify cellular functions via interacting with HIF-1α and NFκB 209. ROS are directly involved in HIF-1a stabilization under hypoxia. ROS signaling via HIF-1a is a key process that is critical in cellular proliferation and angiogenesis 210.

### Mitochondria function in hypoxia

Hypoxia has been regarded as a hallmark of the tumor microenvironment, it happened due to increased oxygen consumption and inadequate oxygen supply in the majority of tumors 211. Hypoxia is associated with poor prognosis and has been demonstrated as a target for cancer therapy 212, 213. It induces changes in gene expression of tumor cells for better adaption to a low oxygen environment. High-proliferation tumor cells rely on glycolysis to obtain ATP for survival. But slowly dividing cells such as cancer stem cells in hypoxic regions are more relying on OXPHOS to obtain ATP and they usually escape from the chemo- and radiotherapy. Hypoxia induces reduced energy production through decreased mitochondrial metabolic activity or altered hypoxia-inducible factor-1 (HIF-1) and peroxisome proliferator-activated receptor gamma coactivator 1(PGC1) dependent mitochondrial biogenesis. Mitochondria are the main consumer of oxygen, in response to hypoxia, mitochondria will moderate their respiratory chain to adjust metabolism, especially in decreasing the activity of TCA cycle 214. By contrast, hypoxia alters mitochondrial fusion and fission, mitophagy and OXPHOS. Super-complexes were formed among complexes I, III, and IV of the ETC to produce minimum superoxide and improve the efficiency of oxygen consumption in hypoxia.

Hypoxia activates mitophagy 215. Chronic hypoxic conditions can deplete the mitochondrial mass by autophagy, resulting in selective clearance of damaged mitochondria in cells 216. The outer mitochondrial membrane protein FUNDC1, a receptor for hypoxia-induced mitophagy, recruits DNM1L/DRP1 to drive mitochondrial fission in response to hypoxic stress to enhance mitophagy 217. The mitochondrial phosphatase PGAM5 dephosphorylates FUNDC1 to activate mitophagy 218. FUNDC1 interacted with LC3 via LC3-binding motif Y(18)xxL(21), and mutation of the LC3-interaction region impaired its interaction with LC3 and the subsequent induction of mitophagy^219^. Knocking down of FUNDC1 prevents autophagy in hypoxia 220. ULK1, one of the core human autophagy-related genes, plays a specific role in mitophagy. ULK1 interacts with FUNDC1, phosphorylating it at serine 17, which enhances FUNDC1 binding to LC3 221. ULK1 deficiency induces an invasive phenotype of breast cancer cells under hypoxia and increases osteolytic bone metastasis. ULK1 depletion attenuates mitophagy ability during hypoxia. phosphorylation of ULK1 by MAPK1/ERK2-MAPK3/ERK1 kinase triggers its interaction with BTRC and subsequent K48-linked ubiquitination and proteasome degradation 222. In addition, Hypoxia can also activate the PINK1/Parkin-mediated mitophagy pathway, induce the excessive proliferation of PASMCs and lead to Pulmonary Artery Hypertension (PAH) 223.

HIF is a master regulator for adaptation to low oxygen content and HIF proteins are key determinants of cellular response to hypoxia 224. They increase the expression of glycolysis and decrease oxygen-dependent ETC complex activity in hypoxia 214. HIF mediates adaptive responses to oxidative stress by nuclear translocation and regulation of gene expression. A small fraction of HIF-1α will translocate to the mitochondria after exposure to hypoxia or H_2_O_2_ treatment. The mitochondrial HIF-1α protects against oxidative stress induced apoptosis and reduces ROS production to maintain mitochondrial membrane potential and reduce the expression of mitochondrial DNA-encoded mRNA in response to hypoxia 225.

Hypoxia also impacts the mitochondrial morphology including cristae structure. More mitochondria are in the fission process to promote mitophagy and keep ROS production in a comparatively low level and maintain decreased respiratory activity 226. The impaired mitochondrial function caused by hypoxia also exacerbates the inflammatory response via metabolic perturbation. Hypoxia induces immune cell dysfunction and leads to an altered metabolic profile 227. Mitochondrial respiration defects in cancer cells cause an increase of NADH, and activation of Akt, thus cancer cells obtain drug resistance and survival advantage in hypoxia. In tumor cells, hypoxia-induced autophagy could reduce oxidative damage and promote cell survival.

## Targeting mitochondria for cancer therapy

Mitochondria dysfunction and oxidative stresses have been found in many diseases including cancers, neurodegenerative disorders, pathogen infections, etc. The mtDNA decrease, OXPHOS dysfunction, and the upregulation of ROS are found in various tumors. The OXPHOS downregulation is associated with poor clinical outcome across most of the cancer types and correlates with a gene signature characteristic of invasive and metastatic tumors. Targeting mitochondria has been reviewed a lot and various inhibitors have been developed (Table 1). Some of the inhibitors described above have moved on to clinical trials due to their relatively stable therapeutic effects.

### Metformin

Metformin has been used as a treatment for diabetes for the past 100 years 228. In recent years, it has been shown in clinical trials to be effective against cancer as well 229. In breast cancer, Metformin reduces tumor cell proliferation by decreasing insulin level since insulin promotes breast cancer cell proliferation, meanwhile, it suppresses tumor progression via inhibiting complex I and PI3K pathway 230. Moreover, Metformin has similar effect in the clinical trial of colorectal cancer, it inhibits mitochondrial complex I and activating AMPK and LKB1 pathways to suppress tumor growth 231. Therefore, Metformin is considered to be a valuable multi-target therapy agent.

### IACS-010759

IACS-010759 is a clinical-grade small-molecule inhibitor of complex I of the mitochondrial electron transport chain. Treatment with IACS-010759 robustly inhibited proliferation and induced apoptosis in models of brain tumors and AML reliant on OXPHOS. IACS-010759 is currently being evaluated in phase 2 clinical trials in relapsed/refractory AML and solid tumors 166.

### Gboxin

Gboxin, a small molecule, emerges as a novel OXPHOS inhibitor that targeting the activity of F0F1 ATP synthase in mitochondria 13. Gboxin inhibits the glioblastoma cancer stem cell growth without affecting the growth of normal cells. In liver cancer, Gboxin inhibits cancer cell ATP production and migration via disrupting the interaction between TOMM34 and ATP5B, it shows synergistic effect with Metformin in liver cancer treatment 232.

### Atovaquone

Atovaquone, an approved antimicrobial drug, has demonstrated anti-cancer potential and ability in clinical trial in treating ovarian cancer recently, it inhibits tumor cell proliferation by reducing ATP production by inhibiting mitochondrial complex III and increasing ROS levels 233. In NSCLC, Atovaquone reduces tumor cells' drug resistance, it inhibits complex III and activates AMPK, leading to cancer cell death 234.

### Dichloroacetic acid

Dichloroacetic acid, a novel anticancer drug, has been proved to have synergistic and inhibitory effects on liver cancer cells in clinical studies 235. Its main target is PDH 236. It inhibits liver cancer cell growth by inhibiting TCA cycle to stimulate AMPK pathway and induce oxidative stress. It has similar effect in glioblastoma 237. In HCC mouse models, it was demonstrated that the combination of Metformin and Dichloroacetic acid had a synergistic effect, Metformin inhibit OXPHOS and upregulates glycolysis in HCC and increases the sensitivity to the PDH kinase inhibitor 238.

### CPI-613

CPI-613 is a potent mitochondria metabolism inhibitor 239. ACC, a key enzyme modulating lipid metabolism, has been demonstrated as a vital target of CPI-613. CPI-613 exhibits anticancer activity in pancreatic cancer cells by triggering ROS-associated apoptosis, accompanied by increased autophagy and repressed lipid metabolism through activating the AMPK signaling 240. Devimistat (CPI-613®) has achieved promising result in Phase I and Phase II study and right now in Phase III study 241.

## Conclusion and perspective

Mitochondrial metabolism is necessary for cancer cell proliferation, tumorigenesis and metastasis. Mitochondria execute mitophagy to clear damaged mitochondria and their fusion and fission dynamics may support tumor stemness and growth. Mutations of mtDNA are commonly found in various tumors, eliminated mutated mtDNA limits tumorigenesis. mtDNA escaped from stressed mitochondria by mPTP- and VDAC-dependent channels to activate the cGAS-STING signaling and gave rise to pro-inflammatory extracellular DNA 283. Mutations of enzymes and metabolites in TCA cycles such as FH and SDH in mitochondria play important roles in tumor invasiveness and metastasis 284. The intermediates in TCA cycles such as succinate, citrate, and NAD^+^ have been demonstrated to possess signaling capacity and to influence immunity 285.

In multiple cancers, the majority of tumor cells especially slow-dividing cancer cells generate ATP via mitochondrial OXPHOS. CSCs are resistant to regular chemo- and radiotherapy, but a great number of them rely on OXPHOS for energy supply, plenty of data highlight the existing connection between CSCs fate and mitochondrial metabolism and lots of inhibitors have been developed to target the complexes or proteins in OXPHOS to reduce CSC viability.

Targeting specific players including both TCA enzymes and OXPHOS-related-genes in mitochondria or mitochondria-related pathways may serve as novel and promising therapeutic approaches for cancers due to the multiple functions of mitochondria in cancer cells. However, the obstacles are there too, mitochondria are not only working in cancer cells but also in normal cells, how to selectively eliminate cancer cells without affecting healthy cells is the biggest challenge. The novel inhibitor Gboxin is one of the trials since its function relies on the high gradient in mitochondria of CSCs. Further investigations are required on the difference of mitochondria between cancer cells and normal ones so that targeting this difference will be a potential selective therapy.

## Figures and Tables

**Figure 4:**
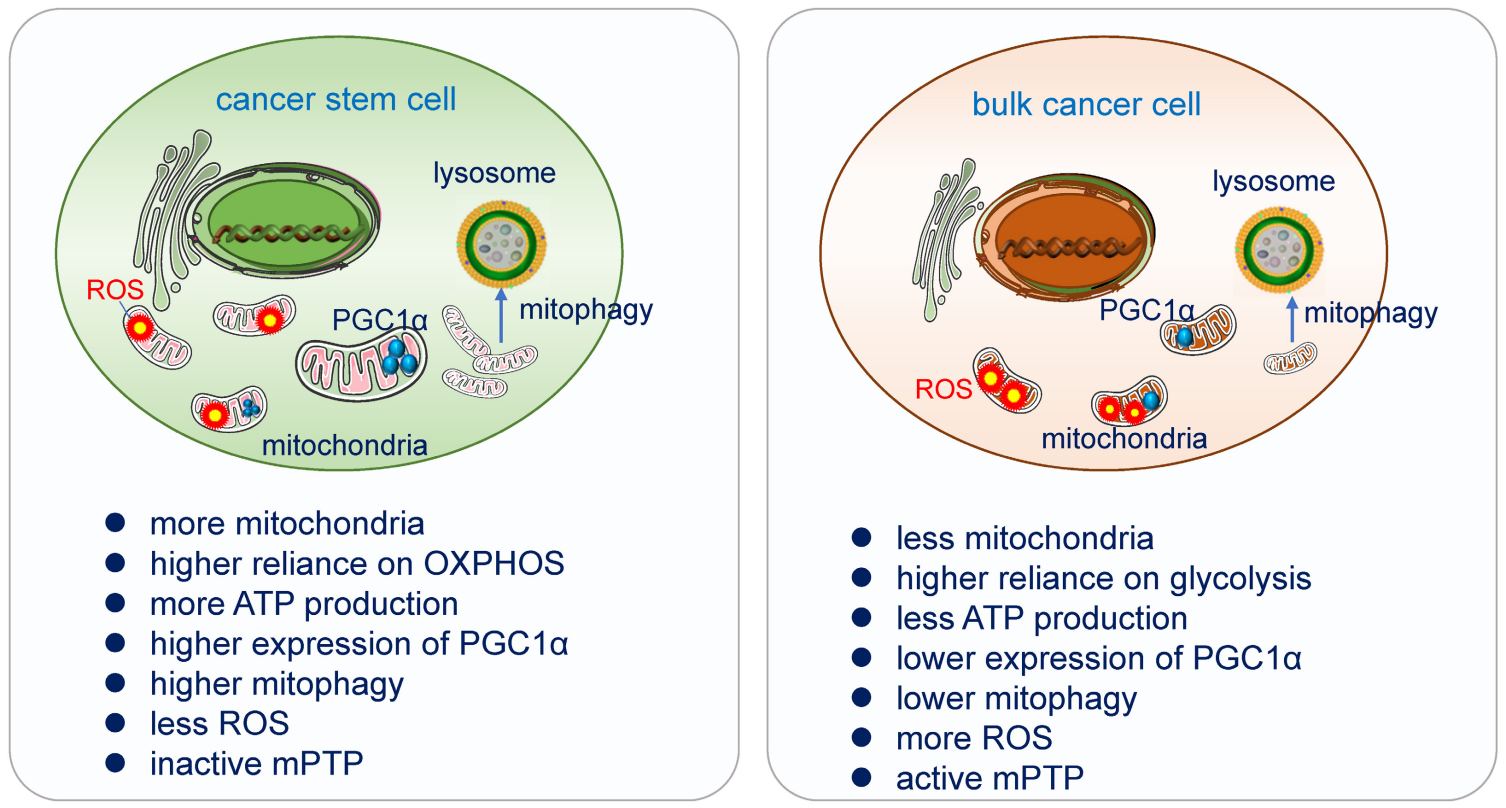
** Differences of mitochondrial functions in cancer stem cell and bulk cancer cell.** Cancer stem cells acquire their energy more from OXPHOS. High expression of PGC-1α enhances mitochondrial biogenesis, strong antioxidant activity and fewer ROS, increases OXPHOS and promotes cancer cells' invasion and metastasis. CSCs possess a more powerful antioxidant defense system which can counteract and scavenge ROS to keep their low ROS level and maintain their stemness and tumorigenesis. Mitophagy is essential in the differentiation and in the acquisition of “stemness” in cancer stem cells, it promotes CSC plasticity for better adaption in TME. CSCs have inactive mPTP and bulk tumor cells have active mPTP 13.

**Table 1:** Inhibitors of mitochondria

Inhibitors	Target	References
Metformin	Complex I	228
IACS-010759	Complex I	166
Phenformin	Complex I	242
ME344	Complex I	243
Fenofibrate	Complex I	244
mIBG	Complex I	245
Pyrvinium	Complex I	246
Canagliflozin	Complex I	247
Pioglitazone	Complex I	248
Rosiglitazone	Complex I	249
Amobarbital	Complex I	250
Nefazodone	Complex I	251
Piericidin A	Complex I	252
mdivi-1	Complex I	253, 254
ginsenoside Rb1	Complex I	255
ASP4132	Complex I	256
Authipyrin	Complex I	257
αTOS	Complex II	258
Lonidamine	Complex II	259
Malonate	Complex II	260
benzenesulfonamide	Complex II	261
Atpenin A5	Complex II	262
2 - alkyl - 4, 6 - dinitrophenols	Complex II	263
Napyradiomycin A1	Complex II	264
Linalool	Complex II	265
picolinamide	Complex III	266
Antimycin A	Complex III	267
Azoxystrobin	Complex III	268
Ametoctradin	Complex III	269
Atovaquone	Complex III	233
Myxothiazol	Complex III	270
Stigmatellin	Complex III	271
mIBG	Complex III	245
Acremonium exuviarum	Complex III	272
Itaconic acid	Complex IV	273
Arsenic trioxide	Complex IV	274
Hydrocortisone	Complex IV	275
6 - hydroxydopamine	Complex IV	276
Cyanide	Complex IV	277
Azide	Complex IV	278
Chrysin	Complex V	279
Oligomycin	Complex V	280
Paroxetine	Complex V	281
Chlorpromazine	Complex V	282
Gboxin	Complex V	13
Baicalein	mPTP	5
